# COVID-19 Outbreak Among Attendees of an Exercise Facility — Chicago, Illinois, August–September 2020

**DOI:** 10.15585/mmwr.mm7009e2

**Published:** 2021-03-05

**Authors:** Frances R. Lendacki, Richard A. Teran, Stephanie Gretsch, Marielle J. Fricchione, Janna L. Kerins

**Affiliations:** ^1^Chicago Department of Public Health, Chicago, Illinois; ^2^University of Illinois at Chicago; ^3^Epidemic Intelligence Service, CDC.

On September 8, 2020, the Chicago Department of Public Health (CDPH) was notified of a potential outbreak of coronavirus disease 2019 (COVID-19) at an exercise facility. COVID-19 cases were identified among 55 (68%) of 81 attendees of in-person classes held during August 24–September 1, 2020, including 49 (60%) cases confirmed by real-time reverse transcription–polymerase chain reaction (RT-PCR) testing and six (7%) probable cases among attendees who had compatible symptoms but negative or no RT-PCR test results. Overall, 43 (78%) attendees with COVID-19 participated in multiple classes while potentially infectious.* Twenty-two (40%) attendees with COVID-19 attended on or after the day of symptom onset. Among 58 exercise class attendees who provided information on in-class behaviors, 44 (76%) reported infrequent mask use, including 32 of 38 (84%) attendees with COVID-19 and 12 of 20 (60%) without COVID-19. The increased respiratory exertion that occurs in the enclosed spaces of indoor exercise facilities facilitates transmission of SARS-CoV-2, the virus that causes COVID-19, in these settings (*1*,*2*). To reduce SARS-CoV-2 transmission in exercise facilities, employees and patrons should wear a mask, even during high-intensity activities when ≥6 ft apart. In addition, facilities should provide engineering and administrative controls including 1) improving ventilation; 2) enforcing consistent and correct mask use and physical distancing (maintaining ≥6 ft of distance between all persons and limiting physical contact, class size, and crowded spaces); 3) reminding infected employees and patrons to stay home and away from others for ≥10 days after symptom onset or, if asymptomatic, after a positive test result, as well as to observe quarantine guidance after close contact with a person with COVID-19 and while awaiting test results; and 4) increasing opportunities for hand hygiene. Conducting exercise activities entirely outdoors or virtually could further reduce SARS-CoV-2 transmission risk. 

## Investigation and Results

During August 24–September 1, 2020, an exercise facility offered four to eight high-intensity indoor classes daily. All classes were held at ≤25% capacity (i.e., 10–15 persons). Mask use, temperature checks, and symptom screenings were required on entry; however, patrons were allowed to remove masks during exercise. Patrons brought their own mats and weights and were stationed ≥6 ft apart. On September 1, a patron notified the facility of receipt of a positive test result. The dates of symptom onset and last exercise class attendance were both August 28. The facility closed for 13 days and informed all attendees of their possible COVID-19 exposure. On September 8, during routine case investigation, CDPH identified a cluster of cases linked to the facility. When CDPH first contacted the facility on September 10, the facility had already notified all attendees (employees and patrons) of potential COVID-19 exposure and learned of 41 patrons with COVID-19–compatible symptoms or positive test results. The facility provided contact information and last attendance date for all persons who had attended classes during August 24–September 1.

Case investigations were conducted using standardized REDCap data collection tools (version 10.3.3; Vanderbilt University). All August 24–September 1 class attendees were contacted for interview during September 14–22. Testing and outcomes data,^†^ social activities,^§^ and in-class behaviors (i.e., mask use and physical distancing) were assessed.

A laboratory-confirmed case was defined as a positive SARS-CoV-2 RT-PCR test result for any facility attendee during August 24–September 15. Attendees with symptoms clinically compatible with COVID-19^¶^ who did not have a positive test result were considered to have probable COVID-19. Self-reported positive test results were confirmed through Illinois’ National Electronic Disease Surveillance System (I-NEDSS). Characteristics of attendees with and without COVID-19 were compared using Fisher’s exact test. Associations between in-class behaviors and COVID-19 case status were estimated using logistic regression.** The primary analyses included probable and confirmed cases. A complete-case sensitivity analysis included only attendees with laboratory-confirmed positive or negative COVID-19 status (i.e., a positive or negative SARS-CoV-2 test result) who also provided information on frequency of in-class mask use and distancing. Analyses were completed using SAS (version 9.4; SAS Institute). This activity was reviewed by CDC and was conducted consistent with applicable federal law and CDC policy.^††^

Among 91 facility attendees (88 patrons and three employees), 10 had neither testing nor interview data available and were excluded. Among the remaining 81 attendees, 55 (68%) COVID-19 cases were identified, including 49 (60%) laboratory-confirmed cases and six (7%) probable cases; all identified cases were among patrons. Seventy-three (90%) attendees were interviewed, including 47 (85%) of 55 with COVID-19. Eight attendees with laboratory-confirmed COVID-19 (16%) were not interviewed.

Sixty-eight (84%) attendees were Chicago residents, 71 (88%) were women, and 72 (97%) were non-Hispanic Black; the median age was 42 years (interquartile range [IQR] = 29–55 years) (Table 1). Among 73 interviewees, 24 (33%) reported medical conditions associated with severe COVID-19 illness^§§^; asthma was the most frequently reported underlying condition, reported by 11 (15%) attendees.

**TABLE 1:** Demographic characteristics, in-class behaviors, and other social exposures among attendees (N = 81) of an exercise facility, by COVID-19 status — Chicago, Illinois, August 24–September 1, 2020

Characteristic	No. (%) of attendees			p-value^†^	OR (95% CI)^§^
	Total (N = 81)	With COVID-19 (n = 55)*	Without COVID-19 (n = 26)		
**Female **	**71 (87.7)**	**48 (87.3)**	**23 (88.5)**	**1.00**	**—**
**Age, yrs, median (IQR)**	**42 (29–55)**	42 (27–57)	41 (29–53)	1.00	—
**Age group, yrs (n = 78)^¶^**					
<18	**1 (1.3)**	1 (1.8)	0 (—)	0.80	—
18–44	**44 (56.4)**	32 (58.2)	12 (52.2)		—
45–54	**21 (26.9)**	13 (23.6)	8 (34.8)		—
55–64	**10 (12.8)**	7 (12.7)	3 (13.0)		—
≥65	**2 (2.6)**	2 (3.6)	0 (—)		—
**Other characteristics**					
Black, non-Hispanic** (n = 74)	**72 (97.3)**	49 (98.0)	23 (95.8)	1.00	—
Underlying medical conditions^††^ (n = 73)	**24 (32.9)**	16 (34.0)	8 (30.8)	1.00	—
No history of smoking^§§^ (n = 68)	**64 (94.1)**	41 (93.2)	23 (95.8)	1.00	—
Pregnant or could be pregnant	**1 (1.4)**	0 (—)	1 (3.8)	1.00	—
**Attendee type**					
Facility patron	**78 (96.3)**	55 (100.0)	23 (88.5)	—	—
Facility employee	**3 (3.7)**	0 (—)	3 (11.5)	—	—
**In-class behaviors**					
Self-reported days of attendance, median (IQR) (n = 53)	**5 (2–8)**	5 (3–7)	3 (1–6)	—	—
Wore a mask during ≤60% of class time^¶¶^ (n = 58)	**44 (75.9)**	32 (84.2)	12 (60.0)	0.06	3.5 (0.9–15.1)
Observed others wearing masks ≤60% of class time (n = 58)	**46 (79.3)**	33 (86.8)	13 (65.0)	0.11	3.5 (0.8–16.6)
Practiced physical distancing ≤60% of class time*** (n = 56)	**4 (7.1)**	3 (8.3)	1 (5.0)	1.00	1.7 (0.1–95.4)
**Other social exposures** ^†††^	**20 (27.4)**	12 (25.5)	8 (30.8)	0.42	—

**Abbreviations:** CI = confidence interval; COVID-19 = coronavirus disease 2019; IQR = interquartile range; OR = odds ratio.

* Attendees with laboratory-confirmed COVID-19 who were not reached for interview (n = 8) were included in analyses of attendance while infectious; dates of positive test result and facility-confirmed last attendance were used.

^†^ p-values from Fisher's exact test were used to compare differences in demographic distributions and in-class behaviors among attendees with COVID-19 versus without COVID-19.

^§^ ORs among attendees with COVID-19 versus without COVID-19 were calculated for mask use, observing others’ mask use, and physical distancing during ≤60% versus ≥61% of exercise class time. Data on frequency of wearing or observing others wearing masks were missing for 17 (30.9%) attendees with COVID-19 and six (23.1%) without COVID-19; physical distancing data were missing for 19 (34.5%) attendees with COVID-19 and six (23.1%) without COVID-19; and data on self-reported classes attended were missing for 19 attendees with COVID-19 (34.5%) and nine without COVID-19 (34.6%).

^¶^ Age was unknown for three (11.5%) attendees without COVID-19.

** Race/ethnicity data were missing for five (9.1%) attendees with COVID-19 and two (7.7%) without COVID-19.

^††^ Reported underlying medical conditions among 73 respondents were as follows (with missing data for eight of 55 attendees with COVID-19): asthma, 11 (15%); hypertension, 10 (13.7%); and diabetes, chronic kidney disease, or prediabetic neuropathy, one (1.4%) each. None of the other underlying medical conditions asked about by interviewers were reported (i.e., chronic heart, liver, or pulmonary disease; seizures; sickle cell disease; or any immunocompromising conditions).

^§§^ Data on smoking status were missing for 11 (20%) attendees with COVID-19 and for two (7.7%) without COVID-19.

^¶¶^ Data on in-class mask use were missing for 17 (30.9%) attendees with COVID-19 and six (23.1%) without COVID-19; data on physical distancing were missing for 19 (34.5%) attendees with COVID-19 and for six (23.1%) without COVID-19.

*** Data on in-class physical distancing were missing for 19 (34.5%) attendees with COVID-19 and for six (23.1%) without COVID-19.

^†††^ Other social exposures reported in the 2 weeks before symptom onset or a positive test result included working outside the home: six (10.9%) attendees with COVID-19, six (23.1%) without COVID-19; dining at restaurants: three (5.4%) attendees with COVID-19, none without COVID-19; attending church: one (1.8%) attendee with COVID-19, none without COVID-19; and other indoor or outdoor activities: nine (16.3%) attendees with COVID-19, three (11.5%) without COVID-19. One (1.8%) attendee with COVID-19 worked in a correctional facility with an ongoing COVID-19 outbreak; one attendee with COVID-19 hosted an indoor gathering with no mask use; one attendee with COVID-19 participated in a group bike ride with no mask use; and one attendee with COVID-19 participated in an indoor party with mask use.

Twenty-two (40%) attendees with COVID-19 reported measured or subjective fever (Table 2). Two (4%) visited an emergency department; one (2%) patient was hospitalized for 8 days. No deaths were reported. Symptom onset dates ranged from August 19 to September 11. Twenty-two (40%) attendees with COVID-19 attended an exercise class on or after the date of symptom onset, including three (5%) who attended on the same day or after they received the positive test result. Overall, 43 (78%) attendees with COVID-19 attended an exercise class during their estimated infectious periods. Attendees with COVID-19 reported participating in a median of five exercise classes (IQR = 3–7); attendees without COVID-19 reported attending a median of three exercise classes (IQR = 1–6).

**TABLE 2:** COVID-19 signs, symptoms, and outcomes among attendees (N = 55) of an exercise facility — Chicago, Illinois, August 24–September 1, 2020

Signs, symptoms, and outcomes	No. (%)*
**Signs and symptoms**	
Headache	38 (69.1)
Loss of taste or smell	33 (60.0)
Myalgia	33 (60.0)
Chills	31 (56.4)
Cough	28 (50.9)
Fever (measured or subjective)	22 (40.0)
Shortness of breath	22 (40.0)
Fatigue	22 (40.0)
Sore throat	13 (23.6)
Diarrhea^† ^	12 (21.8)
Rhinorrhea	11 (20.0)
Nausea or vomiting	10 (18.2)
Congestion	8 (14.5)
Loss of appetite	5 (9.1)
Abdominal pain	5 (9.1)
Confusion	2 (3.6)
**Outcomes**	
Emergency department visit	2 (3.6)
Hospital admission^§^	1 (1.8)
Death	0 (—)

**Abbreviation:** COVID-19 = coronavirus disease 2019.

* Signs, symptoms, and outcome data were unavailable for eight (14.5%) attendees with COVID-19 who were not interviewed.

^† ^Three or more loose, or looser than normal, stools in 24 hours.

^§^ One attendee with COVID-19 was hospitalized for 8 days, without use of oxygen, intubation, or ventilation.

Two attendees with COVID-19 (attendees A and B) reported symptom onset during August 19–20; each attended five classes during August 24–September 1 while symptomatic (Figure). Attendees A and B both received positive SARS-CoV-2 RT-PCR results after the facility closed; both reported mask use ≤60% of the time in class (infrequent mask use).

**FIGURE:**
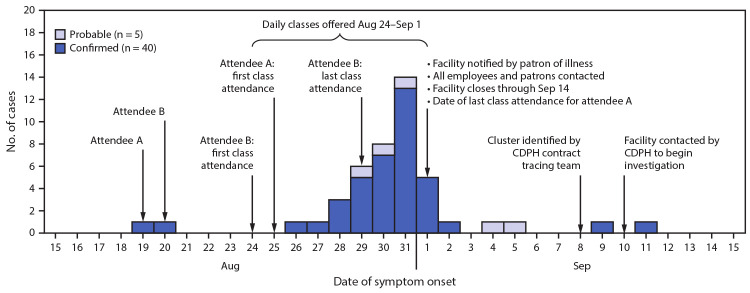
Confirmed and probable COVID-19 cases (n = 45) among attendees of an exercise facility,* by date of reported symptom onset^†^ — Chicago, Illinois, August 19–September 11, 2020 **Abbreviations:** CDPH = Chicago Department of Public Health; COVID-19 = coronavirus disease 2019. *Attendees A and B with COVID-19 each reported attending five classes after symptom onset. ^†^Onset dates were unavailable for 10 (18.2%) of the 55 total cases.

Among 58 (72%) interviewees who provided information on in-class behaviors, including 38 (69%) attendees with and 20 (77%) without COVID-19, infrequent mask use during class was reported more commonly among attendees with COVID-19 (32; 84%) than among those who did not have COVID-19 (12; 60%) (odds ratio [OR] = 3.5; 95% confidence interval [CI] = 0.9–15.1). Twelve attendees with COVID-19 and eight who did not have COVID-19 reported social exposures outside the exercise facility during August 19–September 2 (Table 1). Sensitivity analyses included 32 attendees with positive SARS-CoV-2 RT-PCR test results and 10 with negative results (Supplementary Table; https://stacks.cdc.gov/view/cdc/103076). Findings were similar to those of the primary analysis: 28 (88%) attendees with COVID-19 and six (60%) without COVID-19 reported infrequent mask use during an exercise class; the odds of infrequent mask use were greater (OR = 4.5; 95% CI = 0.6–32.2) among attendees with COVID-19 than among those without COVID-19.

## Public Health Response

After receiving notification of a COVID-19 case in one of its patrons, the exercise facility closed and informed all attendees of possible COVID-19 exposure. CDPH reviewed infection control guidance with the facility, emphasizing the importance of mask use, a 14-day quarantine, isolation, and testing. In addition to following this public health guidance, the facility also asked attendees to provide proof of a negative COVID-19 test result to return to class. At the time of this outbreak, businesses in Chicago were encouraged but not required to report COVID-19 cases. Under CDPH’s revised public health order, city-licensed businesses are now required to report any COVID-19–related suspension of operations and awareness of five or more confirmed COVID-19 cases among employees or patrons.^¶¶^

## Discussion

This outbreak reinforces the need for combined COVID-19 prevention strategies, including universal mask use in public settings when persons are with others who do not live in the same household, especially indoors***; testing of symptomatic persons and those who have been exposed to SARS-CoV-2; self-isolation after symptom onset or a positive COVID-19 test result; and quarantining of persons who have been exposed to SARS-CoV-2 (*3*). Cases were identified among 68% of facility attendees, and CDPH attributed this outbreak to the high proportion of attendees with COVID-19 who participated in class while symptomatic, or asymptomatic and infectious. Most attendees did not wear a mask during exercise class; infrequent mask use when participating in indoor exercise classes likely contributed to transmission. In addition, the potential for infected persons to infect others between their testing date and receipt of test result reinforces the need to quarantine while waiting for a COVID-19 test result and avoid gatherings while unknowingly infectious.

Data on transmission of SARS-CoV-2 in exercise facilities are limited; outbreak reports indicate that increased respiratory exertion might facilitate transmission (*4**–**7*). Clusters of SARS-CoV-2 transmission associated with exercise groups were reported before COVID-19 was declared a pandemic and before mask use was broadly recommended (*5*,*6*). In a more recent outbreak related to an indoor hockey game, only athletic face shields partially covering the nose and mouth were used (*7*).

Although the timing of cases suggests a point-source exposure, none was identified. Most interviewees attended several exercise classes. Some published evidence supports aerosolized transmission of SARS-CoV-2 (*8*), which could have been a contributing factor in this outbreak. Although the facility’s ventilation system was not assessed, inadequate air circulation might have exacerbated transmission in the building, which was not originally designed for exercise classes (*9*).

The findings in this report are subject to at least five limitations. First, because of incomplete interview and testing data, the cases might have been undercounted. Second, not all interviewees reported their class attendance or in-class behaviors, which limited the ability to link cases to particular classes and assess differences between attendees who did and did not have COVID-19. Third, reliance on self-reported behaviors and COVID-19 case status might have introduced recall and social desirability biases. Fourth, nonresponse and the small cohort size limited the precision of effect estimates. Finally, whole-genome sequencing was not performed to assess the phylogenetic relationships among cases linked to the exercise facility, and some attendees with COVID-19 might have acquired different strains of SARS-CoV-2 elsewhere in the community.

The outbreak described in this report occurred despite use of certain COVID-19 mitigation measures. To reduce SARS-CoV-2 transmission in exercise facilities, employees and patrons should wear a mask, even during high-intensity activities (*10*) while ≥6 ft apart.^†††^ In addition, facilities should provide engineering and administrative controls including improving ventilation, enforcing physical distancing, increasing opportunities for hand hygiene, and reminding all employees and patrons to 1) isolate when experiencing COVID-19–like symptoms or after receiving a positive SARS-CoV-2 test result and 2) quarantine after a potential exposure to SARS-CoV-2 and while awaiting test results. Conducting exercise activities entirely outdoors or virtually could further reduce SARS-CoV-2 transmission risk.

**Summary**

**What is already known about this topic?**

Increased respiratory exertion facilitates SARS-CoV-2 transmission; outbreaks linked to indoor activities have been reported.

**What is added by this report?**

In August 2020, 55 COVID-19 cases were identified among 81 attendees of indoor high-intensity classes at a Chicago exercise facility. Twenty-two (40%) persons with COVID-19 attended on or after the day symptoms began. Most attendees (76%) wore masks infrequently, including persons with (84%) and without COVID-19 (60%).

**What are the implications for public health practice?**

To reduce SARS-CoV-2 transmission in fitness facilities, attendees should wear a mask, including during high-intensity activities when ≥6 ft apart. In addition, facilities should enforce physical distancing, improve ventilation, and encourage attendees to isolate after symptom onset or receiving a positive SARS-CoV-2 test result and to quarantine after a potential exposure to SARS-CoV-2 and while awaiting test results. Exercising outdoors or virtually could further reduce SARS-CoV-2 transmission risk.

## Footnotes

* The infectious period was presumed to begin 2 days before symptom onset or positive SARS-CoV-2 test result, whichever was known to be first, and presumed to end ≥10 days after symptom onset or positive test result as long as other symptoms (except loss of taste or smell) were improving and the patient had been fever-free for 24 hours without fever-reducing medication. https://www.cdc.gov/coronavirus/2019-ncov/if-you-are-sick/end-home-isolation.html

† COVID-19 testing and outcomes data included date, result, and location of any SARS-CoV-2 test conducted; date of symptom onset; and recovery. Information on emergency department, intensive care, or other hospital admission was collected, including oxygen administration, ventilation or intubation, and location and length of stay.

§ Social exposures assessed included working outside the home, attending church, visiting someone’s home, attending a party, dining at restaurants, going to bars or music venues, going to gyms other than the exercise facility, gathering with others outdoors, going to a salon, and attending other indoor or outdoor activities.

¶ COVID-19–compatible symptoms assessed included measured fever (≥100.4°F [38°C]), subjective fever, chills, myalgia, rhinorrhea, sore throat, new onset or worsening cough, dyspnea, nausea or vomiting, headache, abdominal pain, diarrhea (three or more loose, or looser than normal, stools in a 24-hour period), and loss of taste or smell. https://wwwn.cdc.gov/nndss/conditions/coronavirus-disease-2019-covid-19/case-definition/2020/

** Crude odds ratios (ORs) with 95% confidence intervals (CIs) were estimated using logistic regression. Odds of wearing masks, observing others wearing masks, and practicing physical distancing in class were compared for 0%–60% of class time versus 61%–100% of class time. Five-point frequency scales used during interviews were dichotomized during analyses because of small cell sizes, which allowed comparison of “most of the time” with “not most of the time.”

†† 45 C.F.R. part 46, 21 C.F.R. part 56; 42 U.S.C. Sect. 241(d); 5 U.S.C. Sect.552a; 44 U.S.C. Sect.3501 et seq.

§§ Underlying medical conditions assessed were asthma, chronic heart, kidney, liver or pulmonary disease, diabetes, hypertension, obesity, seizures, sickle cell disease, and any immunocompromising conditions.

¶¶ Since October 1, 2020, Public Health Order 2020–2 has mandated that city-licensed businesses report to CDPH 1) any suspension in operations because of COVID-19 cases among employees or patrons and 2) awareness of five or more employees or patrons with positive COVID-19 test results within a 14-day period. https://www.chicago.gov/content/dam/city/sites/covid/health-orders/CDPH%20Order%202020-2%203rd%20Amended%20FINAL%209.30.20_AAsigned.pdf

***
https://www.cdc.gov/coronavirus/2019-ncov/prevent-getting-sick/how-to-wear-cloth-face-coverings.html

††† Per CDC guidance, persons who are unable to wear a mask because of difficulty breathing during high-intensity activities must choose a location with greater ventilation and air exchange (e.g., outdoors versus indoors) where it is possible to keep ≥6 ft from others during activity. https://www.cdc.gov/coronavirus/2019-ncov/prevent-getting-sick/cloth-face-cover-guidance.html
